# Importance of early arthrocentesis in temporomandibular joint closed lock by disc displacement without reduction. A case review

**DOI:** 10.4317/jced.62767

**Published:** 2025-06-01

**Authors:** Jordi Borrás-Ferreres, Cosme Gay-Escoda

**Affiliations:** 1 DDS. MSc. Private practice of Oral Surgery and Implantology, Benicarló (Castellón), Spain; 2 MD, DDS, MSc, PhD, EBOS, OMFS. Chairman and Professor of the Department of Oral and Maxillofacial Surgery, Faculty of Medicine and Health Sciences, School of Dentistry, University of Barcelona. Director of the Master degree program in Oral Surgery and Implantology, EFHRE International University/FUCSO, Barcelona. Founder/Researcher of the IDIBELL Institute. Head of the Department of Oral and Maxillofacial Surgery and Implantology, Teknon Medical Center, Barcelona, Spain

## Abstract

Arthrocentesis (joint lysis and lavage) and hydraulic distension with manipulation of the temporomandibular joint have been described as effective options for reducing joint pain and improving function in patients with limited mouth opening (closed lock) due to disc displacement without reduction, fundamentally in the acute phase of the disorder. However, the efficacy and scientific basis of such treatment have not been validated to date. As a result, the most frequent management strategies tend to be more conservative and mainly focus on the use of nonsteroidal antiinflammatory drugs and/or muscle relaxants, occlusal splints and physiotherapy. The present study was carried out to assess the efficacy of early arthrocentesis in improving mouth opening and reducing joint pain in a woman with a 10-day history of acute closed lock due to left temporomandibular joint disc displacement without reduction.

** Key words:**Arthrocentesis, early use, closed lock, disc displacement without reduction, limited mouth opening, temporomandibular joint.

## Introduction

Temporomandibular joint (TMJ) closed lock is typically the result of a normally anterior or anteromedially displaced joint disc that is not reducible and acts as an obstacle against anterior displacement of the mandibular condyle (1). The associated clinical signs are restricted anterior translation of the mandible, the absence of joint sounds (clicks), deflection towards the affected side on opening the mouth, limitation of lateral movement towards the contralateral side and restricted protrusion, with the mandible displacing towards the affected side. Acute cases are characterized by joint pain in response to palpation and on opening the mouth (2). The diagnostic imaging technique of choice is magnetic resonance imaging (MRI), where closed lock is seen as disc displacement without reduction (DDwoR) with the mouth open (1,3).

Conservative management is the first choice; in cases of “acute” closed lock it seeks to resolve the limitation of mouth opening by reducing the joint disc through manual or instrumental manipulation (physiotherapy), proving successful in about 18% of the cases (4). If this approach fails, management focuses on educational therapy with self-care measures, with the use of thermotherapy and a soft diet, as well as the prescription of occlusal splints, drug treatment (nonsteroidal antiinflammatory drugs [NSAIDs] and/or muscle relaxants) and mandibular physiotherapy (5-8). These conservative treatments, being the majority, have yielded results similar to those obtained with minimally invasive surgery (MIS) of the TMJ, such as arthrocentesis and arthroscopy (9-11). However, conservative management is spread out over a long period of time, which in some cases places the patient at an increased risk of developing chronic pain and adverse drug effects, and moreover results in increased economic costs and chronification of the disorder (12-14).

Among the MIS options, arthrocentesis (joint lysis and lavage) and hydraulic distension with manipulation of the TMJ have been shown to be effective in immediately reducing joint pain and increasing the range of mouth opening in patients with “acute” closed lock of the TMJ due to DDwoR (15,16). According to the literature, the success rate of arthrocentesis in these cases is over 75% (16,17).

The present study was carried out to assess the efficacy of arthrocentesis in improving mouth opening and reducing joint pain in a woman with a 10-day history of “acute” closed lock due to left TMJ DDwoR, and to assess the benefits of early arthrocentesis versus the more conservative management options.

## Case Report

**Figure 1:**
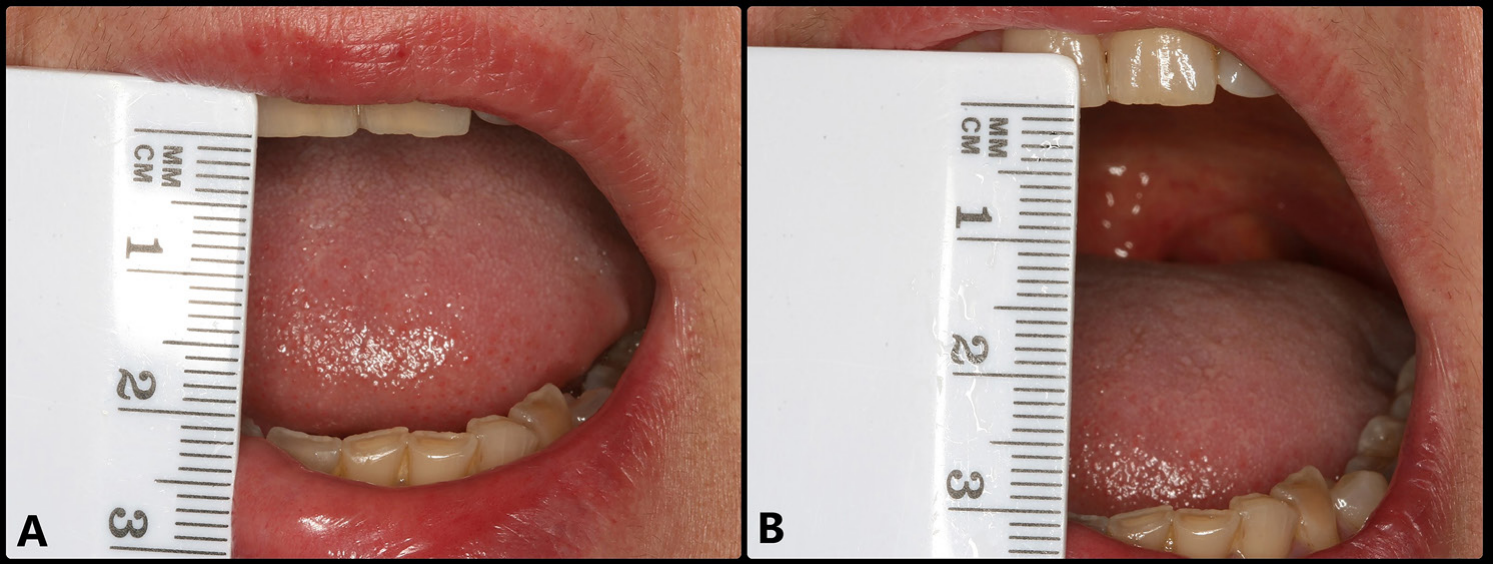
Baseline view showing disc displacement without reduction and limited opening of the mouth. A: Opening of the mouth limited to about 20 mm. B: Opening of the mouth increased to 30 mm after anesthesia of the auriculotemporal nerve.

A 54-year-old woman presented with manifestations suggestive of left TMJ DDwoR, during the last 5 days. She presented severe and painful mouth opening limitation (20 mm), with deflection towards the affected side (Fig. 1A). She explained that closed lock occurred suddenly, accompanied by a strong click while she was chewing gum. The patient suffered bruxism, and since 2015 she had been prescribed with an occlusal splint which she never wore. Joint distraction was carried out in an attempt to reduce the disc, but without success. Early arthrocentesis was therefore recommended to resolve the restricted mandibular movements and joint pain as soon as possible.

**Figure 2:**
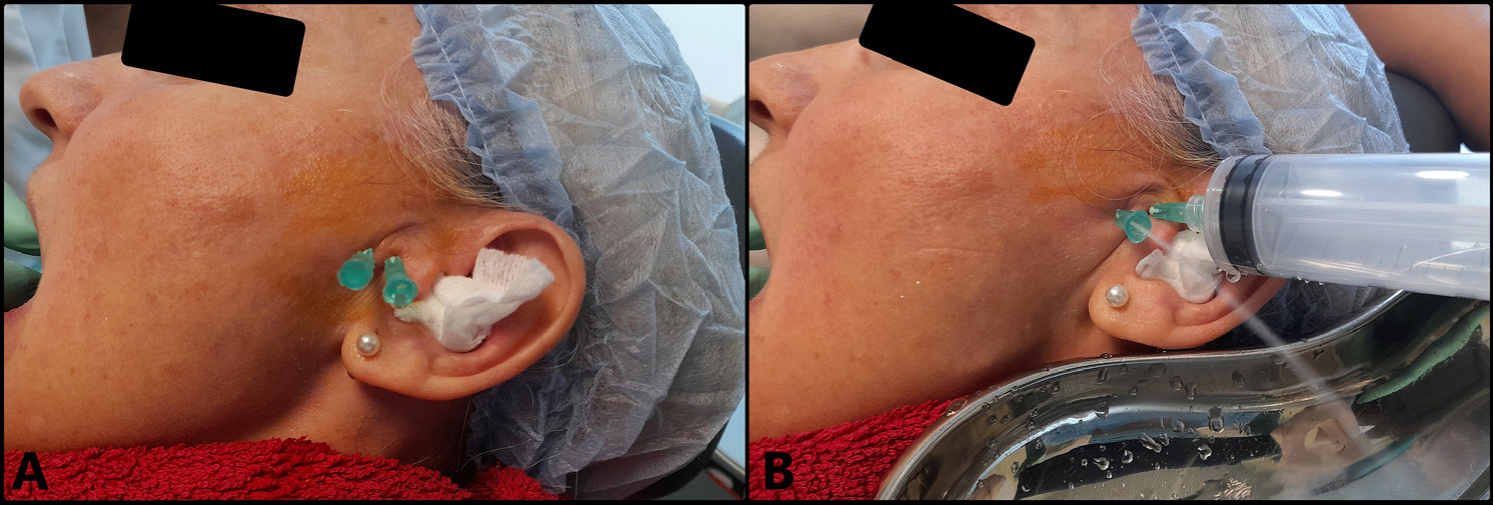
Arthrocentesis. A: Needles positioned in the superior joint space. B: Intraarticular lysis and lavage.

**Figure 3:**
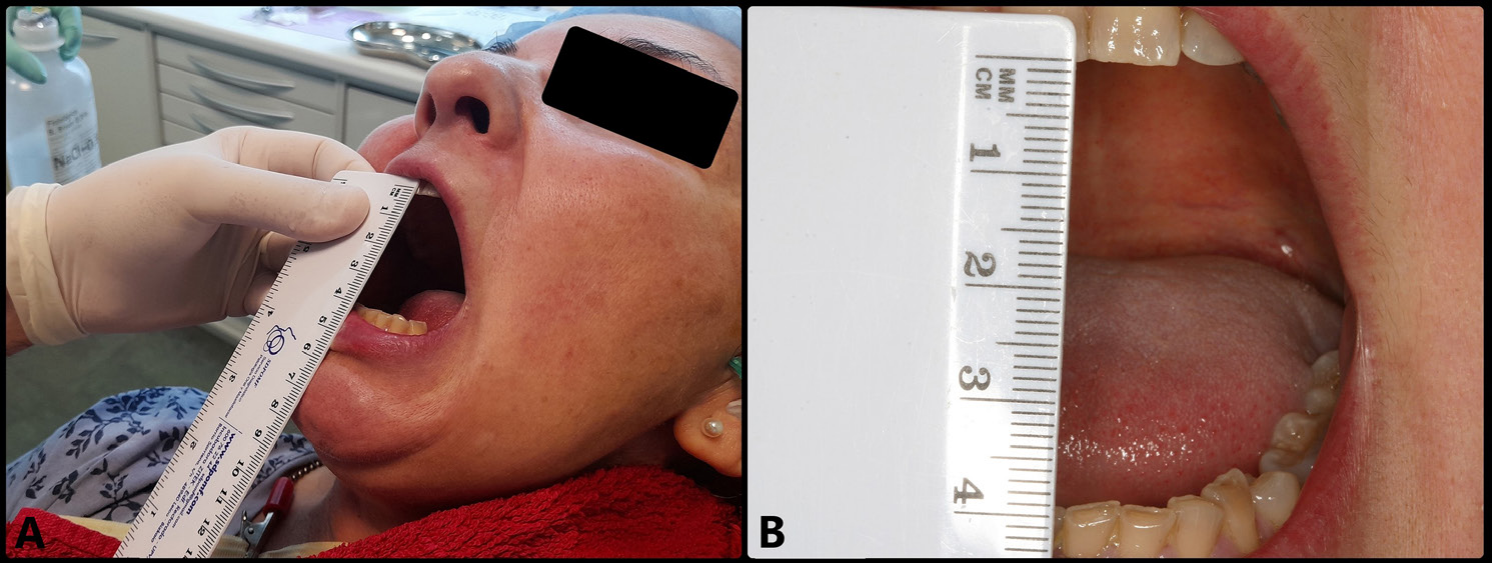
Post-treatment view showing disc displacement without reduction and no limitation of opening of the mouth. Following arthrocentesis, maximum opening was about 40 mm.

MIS (arthrocentesis) was performed 5 days later (10 days after onset of the clinical manifestations), under local anesthesia using articaine with epinephrine 1:200,000 (Ultracain®, Laboratorios Normon, Madrid, Spain) to infiltrate the territory of the auriculotemporal nerve and anesthetize the region of the joint. Following anesthesia, mouth opening range was seen to be 30 mm. A 21G intramuscular needle 25 mm in length was inserted to access the superior joint space (SJS) through its posterior recess, with maximum mouth opening. This needle was used to inject the solution used for lavage, while a second intramuscular 21G needle measuring 25 mm in length and positioned 5 mm anterior and inferior to the first needle was used to evacuate the solution (Fig. 2A). Joint lavage was performed with 120 ml of Ringer lactate (Braun, Barcelona, Spain) (Fig. 2B). During lavage, evacuation of the solution was momentarily paused with a finger in order to increase intra-articular distension, reducing its negative pressure. The first 60 ml were used with the mouth of the patient open, and the remaining 60 ml were administered while the patient performed active opening, closing and lateralization movements. At the end of the procedure, 1 ml of Hylan G-F 20 (Synvisc®, Genzyme Biosurgery, Ridgefield, NJ, USA) was injected as a cartilage-protecting measure thanks to its analgesic, lubricating and antiinflammatory effects. The result was excellent, with instantaneous and complete elimination of the joint closed lock, maximum mouth opening of 40 mm, improvement of the mandibular movements, and disappearance of the joint pain, (Fig. 3).

## Discussion

In “acute” joint lock, the most frequent and conservative initial treatment options seek to resolve the limitation by reducing the disc through manual manipulation and other physiotherapy techniques, with a success rate of about 18% (4). If such measures fail, management focuses on educational therapy with self-care measures, with the use of thermotherapy and a soft diet, as well as the prescription of occlusal splints, and drug treatment (nonsteroidal antiinflammatory drugs [NSAIDs] and/or muscle relaxants) (8). Furthermore, since immobility of the disc seriously affects lubrication, with the development over time of strong adherences that hinder its displacement (18), it is always advisable to prescribe physiotherapy including active and passive movements of the mandible (5-7). In a study on the efficacy of physiotherapy, Nicolakis *et al*. (5) reported a decrease in pain and improvement of the range of mandibular movement of 80% and 75%, respectively, as well as arrested joint degeneration. The authors concluded that physiotherapy is the most effective and useful conservative strategy for treating internal derangement of the TMJ. However, the efficacy of such treatment is dependent on the age of the patient and the duration of closed lock (7). Furthermore, the technique must be applied correctly, exercising increased mandibular condyle rotation and lateralization towards the side not affected by DDwoR (6).

These conservative measures are the predominant treatment strategies, and are reported to yield results similar to those of TMJ MIS techniques (9-11). Ritto *et al*. (11), in a recent double-blind randomized clinical trial, compared the efficacy of conservative management and arthrocentesis for reducing joint pain and improving opening of the mouth. The study involved 60 patients divided into four groups (conservative; conservative + medication; arthrocentesis; and arthrocentesis + medication). The authors recorded improvement of the clinical parameters in all the groups, with no statistically significant differences between them (conservative management versus arthrocentesis). Nevertheless, non-surgical conservative management is spread out over a long period of time, which in some cases places the patient at an increased risk of developing chronic pain and adverse drug effects, and moreover results in increased economic costs and chronification of the disorder (12-14).

A randomized controlled trial with a follow-up period of over 5 years carried out by Tang *et al*. (14) found arthrocentesis as first treatment for joint pain to be superior to conservative management, allowing the immediate start of joint recovery. In their study, 26% of the patients subjected to conservative management (soft diet, physiotherapy and an occlusal splint) subsequently required arthrocentesis, while only 6% of the patients subjected to initial early arthrocentesis required repetition of the technique or the intra-articular injection of methylprednisolone. Mandibular function was similar in both groups, however. A previous simple-blind prospective study by Diraçoğlu *et al*. (19) reported similar results: both the patients with DDwoR subjected to conservative treatment (soft diet, thermotherapy and physiotherapy) and those subjected to arthrocentesis showed improvement of mandibular movement and pain over 6 months of follow-up. However, the arthrocentesis group showed significantly faster pain reduction. Another study conducted by Sembronio *et al*. (20) in 33 patients with closed lock due to DDwoR found arthrocentesis combined with a soft diet, physiotherapy and an occlusal splint to be effective in improving function and reducing pain in all the patients. However, the outcomes were better in the acute cases than in chronic patients. Thus, reconsideration of the existing therapeutic strategies in favor of MIS techniques could be of benefit to patients with very severe pain.

The importance of early MIS techniques is warranted by a recent meta-analysis published by Al-Moraissi *et al*. (21), where such treatment was found to be more beneficial than conservative management over the short (≤ 5 months) and middle term (6 months – 4 years). A previous retrospective cohort study of patients with inflammatory/degenerative TMJ disorders published by Israel *et al*. (22) found the outcomes in terms of decreased pain and improved opening of the mouth to be better when arthroscopy was performed early. The results of these studies support a paradigm shift in the management of internal derangement of the TMJ, with the recommendation not to prolong conservative treatment too long if good results are not obtained quickly, and to perform MIS techniques as soon as possible in such cases. Indeed, may even be seen as a first-line choice in patients with very severe or chronic joint disorders.

In contrast to the traditional concept of first exhausting all possible conservative management options, MIS techniques could be regarded as an effective first-line treatment or as an early management strategy to be performed as soon as initial conservative treatment is seen to offer no clear benefit, or in patients with intense joint pain (14,19,22). Although the symptoms and function in patients with DDwoR tend to improve spontaneously over time, the degree of improvement depends on the time from onset of the disorder (23,24). Yura (25), in a study of the natural course of DDwoR from the “acute” phase (less than one week), found that 95% of the patients experienced spontaneous improvement of opening of the mouth and joint pain after three months – this being regarded as the time limit for considering arthrocentesis in order to quickly reduce the pain and facilitate movement of the mandible and joint disc if no such patient improvement is observed.

Arthrocentesis is very effective in eliminating inflammation and pain mediators, resulting in an immediate and significant reduction of the joint pain that limits mandibular movements and makes it difficult for the patient to perform the required physiotherapy exercises (14,26). The presence of pain limiting opening of the mouth was noted in our patient, with opening improving from 20 mm to 30 mm following the anesthetic effect. In addition to eliminating joint pain, arthrocentesis reduces intra-articular pressure and the viscosity of the altered synovial fluid, and may even disrupt small adherences thanks to hydraulic distension of the SJS – thereby reducing the friction between the joint surfaces that hinders disc displacement (27). Following joint distension and lavage, our patient improved opening of her mouth to 40 mm.

The ultimate aim of MIS techniques is to allow the condyle to push and deform the anteriorly displaced disc to achieve greater translation movement, with opening of the mouth beyond 35 mm (2,28). In order to maintain the improvement achieved, it is important for the patient to wear an occlusal splint, which reduces intra-articular pressure and relaxes the chewing muscles. It is also important to plan early physiotherapy exercises to preserve disc mobility and prevent further joint locks resulting from functional inactivity (5-7,29,30).

In our opinion, the cost-benefit ratio of arthroscopy is debaTable. The systematic review and meta-analysis conducted by Al-Moraissi (31) found arthroscopy to be superior to arthrocentesis in managing pain and improving function in internal derangement of the TMJ. However, these same authors suggested that this could be due to the fact that the former technique is characterized by greater lavage and distension of the joint space, resulting in the rupture and elongation of more adherences, with a decrease in friction between the joint surfaces. In addition, the general anesthesia used contributes to secure maximum muscle relaxation. A previous study published by Clark *et al*. (32) found 83% of the patients with closed lock to recover mobility after simple arthroscopic lysis and lavage; they therefore considered that repositioning of the joint disc through open or arthroscopic surgery should be re-evaluated. On the other hand, Sanders *et al*. (33), in 15 patients with joint hypomobility secondary to sagittal osteotomies of the mandible treated with simple arthroscopic lysis and lavage, reported a significant mean improvement of 11 mm in opening of the mouth, and all the patients experienced important pain relief. It was therefore suggested that surgical manipulation of the joint tissues was not necessary.

In agreement with White (34), we consider that most of the success of arthroscopy in treating closed lock is attribuTable to lavage rather than to surgical instrumentation. As a result, the success afforded by arthrocentesis is similar to that of arthroscopic lysis and lavage. In fact, in a retrospective clinical study carried out by González-García (35) on the effect of arthroscopy in 344 joints with chronic DDwoR, no significant differences were observed in terms of diminished pain and increased opening of the mouth between simple arthroscopy (lysis and lavage) and the technique involving surgical manipulation of the joint tissues. The advantages of arthrocentesis include few complications compared with arthroscopy, its low morbidity, simplicity and low cost, since no general anesthesia with hospital admission is required (36). Indeed, it may represent the TMJ surgical treatment with the best cost-benefit ratio of all the techniques currently available.

On the other hand, in 2010 Escoda-Francolí *et al*. (37) published a systematic review that reported a type A recommendation (according to the SORT criteria) (38) in favor of the intra-articular injection of hyaluronic acid in the multidisciplinary management of TMJ dysfunction. As regards final complementing of arthrocentesis and arthroscopy with the injection of drugs to improve the treatment outcomes, hyaluronic acid has type B recommendation (recommended, but further high-quality studies are needed) (39). In this respect, Hylan G-F 20 is a hyaluronic acid derivative with mechanical properties superior to those of synovial fluid and hyaluronate solutions, with cartilage-protecting and lubricating effects that help reduce joint pain and secure increased mandibular mobility ranges (40). In contrast, the use of platelet-rich plasma and plasma rich in growth factors has type C recommendation (not recommended, due to a lack of sufficient scientific evidence) (41). Furthermore, in all patients with craniomandibular disorders, and especially in those with internal derangements of the TMJ, we recommend the oral administration of cartilage protectors (glycosamine sulfate and chondroitin sulfate), since they contribute to improve both pain control and opening of the mouth, thanks to their action on different inflammatory biomarkers in synovial fluid (42).

## Conclusions

Arthrocentesis rapidly eliminates the pain in cases of arthralgia secondary to DDwoR, allowing patients to reduce their need for drug treatments and facilitating the subsequent physiotherapy exercises. Compared with the more conservative treatment options, arthrocentesis affords important savings in terms of resources and healthcare costs, and helps avoid chronification of the disorder. It therefore should be performed early and as soon as the initial conservative management measures no longer show any clear benefit for the patients.

## Data Availability

The datasets used and/or analyzed during the current study are available from the corresponding author.
